# Association of serum bilirubin level with lung function decline: a Korean community-based cohort study

**DOI:** 10.1186/s12931-018-0814-z

**Published:** 2018-05-23

**Authors:** Ah Young Leem, Ha Yan Kim, Young Sam Kim, Moo Suk Park, Joon Chang, Ji Ye Jung

**Affiliations:** 1 Division of Pulmonology, Department of Internal Medicine, Institute of Chest Disease, Severance Hospital, Yonsei University College of Medicine, 50-1 Yonsei-ro, Seodaemun-gu, Seoul, 120-752 Republic of Korea; 2 Biostatistics Collaboration Unit, Department of Biomedical Systems Informatics, Yonsei University College of Medicine, Seoul, Republic of Korea

**Keywords:** Bilirubin, Lung function, Biomarker

## Abstract

**Background:**

Bilirubin has been reported to be associated with respiratory diseases due to its antioxidant action. We aimed to evaluate the relationship between serum bilirubin concentration and annual lung function decline in the Korean general population.

**Methods:**

The study included 7986 subjects aged 40–69 years from the Ansung-Ansan cohort database I (2001–2002)–III (2005–2006). We analyzed the relationships between serum bilirubin level and forced expiratory volume in 1 s (FEV_1_), forced vital capacity (FVC), FEV_1_/FVC, and mean forced expiratory flow between 25 and 75% of FVC (FEF_25–75%_) at baseline, as well as the annual average changes in these lung parameters.

**Results:**

The FEV_1_, FVC, and FEF_25–75%_ were significantly associated with serum bilirubin levels after adjustment for age, sex, body mass index (BMI), and smoking status (all *P* < 0.001). When stratified according to smoking status, these relationships were significant in never-smokers. Additionally, serum bilirubin level was negatively associated with the annual decline in FEV_1_ and FVC, and positively associated with the annual decline in FEV_1_/FVC after adjustment for age, sex, BMI, baseline lung function, and smoking status (all *P* < 0.001).

**Conclusions:**

We found significant associations of serum bilirubin levels with FEV_1_, FVC, and FEF_25–75%_ in the general population, especially in never-smokers. Moreover, serum bilirubin levels were related with the annual decline in FEV_1_, FVC, and FEV_1_/FVC ratio.

## Background

Serum total bilirubin is routinely measured to identify hepatobiliary and hemolytic diseases. Bilirubin is an end-product of heme degradation that has raised considerable interest over the last decade [1]. The benefits of elevated serum bilirubin are supported by animal and in vitro experiments showing antioxidant and anti-inflammatory properties [2]. In clinical studies, increased serum bilirubin has been associated with decreased risk for myocardial infarction, coronary artery disease, and stroke [3, 4] as well as lower incidence of lung cancer, chronic obstructive lung disease, and lung cancer mortality [5, 6]. It has been suggested that bilirubin might have protective effects in tissues exposed to the outer environment, such as the lungs, possibly by counteracting subclinical inflammation [3–6].

Only few previous studies have reported the relationship between serum bilirubin level and lung function parameters [7, 8]. In the study by Curjuric et al., higher bilirubin levels were associated with higher forced expiratory volume in 1 s (FEV_1_)/forced volume capacity (FVC) ratio and forced expiratory flow at 25–75% (FEF_25–75%_) in the Swiss general population [7]. In the study by Apperley et al., serum bilirubin was positively related to FEV_1_ and negatively related to the annual decline in FEV_1_ in subjects with chronic obstructive pulmonary disease (COPD) with mild to moderate airflow limitation [8]. However, the relationships between bilirubin levels and decline in lung function parameters in the healthy general population have not been reported before. We aimed to evaluate the relationship between serum bilirubin concentration and annual lung function decline in the Korean general population.

## Methods

### Study population

The Ansung-Ansan cohort study is an ongoing prospective study that was started in 2001 with support from the National Genome Research Institute (Korea Centers for Disease Control and Prevention, Cheongju, Korea). The study is a part of the Korean Genome and Epidemiology Study, a large community-based epidemiologic survey to investigate chronic disease in Koreans. Detailed information on the study design and procedures has been published previously [9, 10]. Each study comprised a population-based sample of male and female Koreans aged 40–69 y and from the same ethnic background, but cohort members were enrolled at the following 2 different sites: Ansan, which is an urban community with a population of 555,000, and Ansung, which is a rural community including 133,000 residents, on the basis of the 2000 census. To enroll members for each cohort, the most efficient method was used on the basis of knowledge about characteristics of each community. For enrollment at the Ansan site, 10,957 eligible subjects were identified by telephone contact on the basis of a 2-stage cluster-sampling method with the information of a governing district called Dong and demographic characteristics. Similarly, Ansung members were recruited from 5 of 11 governing districts called Myon by using a cluster-sampling method, and as a result, 7192 eligible subjects were identified by mail or telephone contact and a door-to-door visit. For the baseline health examination from 18 June 2001 to 29 January 2003, 5020 participants (2523 men and 2497 women) from Ansan and 5018 (2239 men and 2779 women) from Ansung visited the Korea University Ansan Hospital and the Ajou University Medical Center, respectively. Cohort members were followed up biennially with a scheduled site visit for similar interviews and health examinations (I: 2001–2002, II: 2003–2004, III: 2005–2006).

**Fig. 1:**
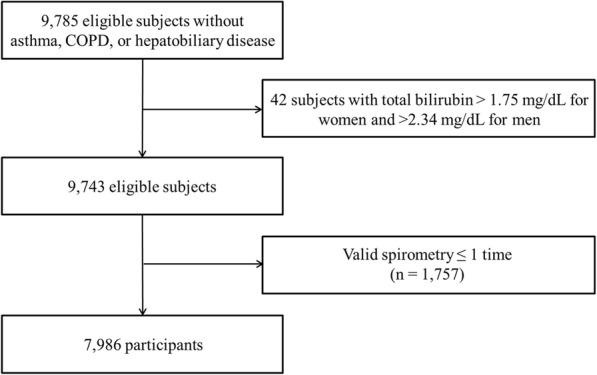
Flow chart of participant selection

In these subjects, initial data were obtained from 9785 subjects aged 40–69 years who participated in Ansung-Ansan cohort I (2001–2002) and had valid lung function measurements without previous history of asthma, COPD, or hepatobiliary disease. Follow-up examinations are conducted biennially. Data from a baseline survey and two subsequent biennial surveys (I–III: 2001–2006) were analyzed in our study. Of the 9785 subjects in the study, 42 participants with total bilirubin concentrations > 1.75 mg/dL for women and > 2.34 mg/dL for men were excluded from the analysis. Finally, 7986 who underwent pulmonary function testing two times or more during I–III were used as the study population in this study (Fig. 1). The median follow-up duration was 3.6 years.

### Bilirubin measurement

At baseline, venous blood samples were collected from eligible participants who originally consented, on the same day of lung function tests. Serum bilirubin was quantified by biochemical assays, performed by a central laboratory (Seoul Clinical Laboratories, Seoul, Korea) [11]. As described above, total bilirubin concentrations > 1.75 mg/dL for women and > 2.34 mg/dL for men were excluded from analysis. These limits represent concentrations 1 standard deviation (SD) above the mean serum total bilirubin associated with the most common variant of Gilbert syndrome, a benign hereditary cause of indirect hyperbilirubinemia [5].

### Lung function measurement

Lung function was measured by spirometry (VMAX2130, SensorMedics Corporation, Yorba, CA, USA) at every visit (at baseline, and at the first and second follow-up visits).

Lung function decline was calculated as the difference in FEV_1_ measurements between the first and last measurement in each time interval divided by the number of years between measurements.

### Definitions

We considered ‘never-smokers’ those individuals who declared that they never smoked before the study, ‘former smokers’ those smokers who declared that they had smoked before and stopped before the study, and ‘continuous smokers’ those smokers who declared active smoking in the study period.

### Statistical analysis

We analyzed the relationships between bilirubin and FEV_1_, FVC, FEV_1_/FVC, and FEF_25–75%_ at baseline, and the annual average decline of these lung parameters. Participants were divided into quintiles of bilirubin concentration, and baseline characteristics were compared using analysis of variance or Cochran-Mantel-Haenszel’s test for trend. We performed the analysis using linear regression and bilirubin as a continuous variable rather than in quintiles. Linear regression with adjustment for age, sex, body mass index (BMI), and smoking status was used to examine the relationship between bilirubin and lung function parameters such as FEV_1_, FVC, FEV_1_/FVC ratio, and FEF_25–75%_. The linear mixed model was used to evaluate the relationship between bilirubin and repeated-measured FEV_1_, FVC, FEV_1_/FVC, and FEF_25–75%_. All analyses were performed using R version 3.1.3 (http://www.R-project.org) and SAS (version 9.4, SAS Inc., Cary, NC, USA).

## Results

### Baseline characteristics

The demographics and clinical characteristics of the 7986 participants, stratified by quintiles of bilirubin concentration, are presented in Table 1. Age was negatively related to serum bilirubin concentration (*P* < 0.001). The proportion of male gender was positively related to serum bilirubin (*P* < 0.001). The proportion of continuous smokers and former smokers was increased while that of never-smokers was decreased toward higher quintiles of bilirubin concentration (*P* < 0.001). Lung function parameters of FEV_1_, FVC, FEV_1_/FVC, and FEF_25–75%_ were positively related to serum bilirubin level (all *P* < 0.001) (Table 1).

**Table 1:** Baseline characteristics of the study participants by quintiles of bilirubin concentration

Parameters	Quintile I	Quintile 2	Quintile 3	Quintile 4	Quintile 5	*P* for Trend
	(*n* = 1534)	(*n* = 1618)	(*n* = 1611)	(*n* = 1600)	(*n* = 1623)	
Bilirubin, umol/L	0.29 ± 0.05	0.42 ± 0.03	0.53 ± 0.04	0.68 ± 0.05	1.04 ± 0.26	< 0.001
Age, y	53.9 ± 9.0	53.1 ± 8.8	51.7 ± 8.6	51.1 ± 8.4	49.9 ± 8.4	< 0.001
Sex, male	401(26.14)	616(38.07)	750(46.55)	959(59.94)	1136(69.99)	< 0.001
BMI	24.5 ± 3.2	24.7 ± 3.2	24.7 ± 3.2	24.8 ± 2.9	24.6 ± 2.9	0.539
Smoking (baseline)						< 0.001
Continuous smoker	311(20.27)	400(24.72)	409(25.39)	454(28.38)	422(26.00)	
Former smoker	117(7.63)	169(10.44)	241(14.96)	307(19.19)	452(27.85)	
Never smoker	1106(72.10)	1049(64.83)	961(59.65)	839(52.44)	749(46.15)	
Pack-Y smoking	24.9 ± 18.6	24.6 ± 17.5	23.4 ± 15.8	24.5 ± 17.3	20.1 ± 15.8	< 0.001
Lung function						
FEV_1_, L	2.62 ± 0.61	2.78 ± 0.66	2.93 ± 0.67	3.06 ± 0.69	3.20 ± 0.68	< 0.001
FVC, L	3.30 ± 0.78	3.51 ± 0.85	3.70 ± 0.97	3.84 ± 0.87	4.00 ± 0.83	< 0.001
FEV_1_/FVC, %	79.9 ± 7.6	79.7 ± 7.6	79.8 ± 7.5	80.2 ± 7.3	80.3 ± 7.3	0.044
FEF_25–75%,_ L/s	2.75 ± 1.02	2.87 ± 1.03	3.05 ± 1.10	3.20 ± 1.16	3.46 ± 4.17	< 0.001

Notes: Data are presented as the number (%) or the mean ± standard deviation

Abbreviations: *BMI* body mass index, *FEV*_*1*_ forced expiratory volume in 1 s, *FVC* forced vital capacity, *FEF*_*25–75%*_ forced expiratory flow between 25 and 75% of vital capacity

### Associations of bilirubin with lung function parameters

Table 2 shows the relationship between serum bilirubin and lung function according to smoking status, adjusted for covariates. When stratified by smoking status and adjusted for age, sex, and BMI, a positive relationship between bilirubin quintiles and FEV_1_ was present in continuous smokers (*P* = 0.048) and never-smokers (*P* < 0.001). When bilirubin was analyzed as a continuous variable, the positive relationship between bilirubin concentration and FEV_1_ was present only in never-smokers (*P* < 0.001). Lung function of FVC was positively related with bilirubin quintiles (*P* = 0.018) and bilirubin level as continuous variable (*P* = 0.012) only in never-smokers. Positive relationship was observed between bilirubin quintile and FEV_1_/FVC ratio in never-smokers (*P* = 0.026). However, when bilirubin was analyzed as a continuous variable, the effect of bilirubin on FEV_1_/FVC ratio was not statistically significant. There was a significant positive relationship between bilirubin and FEF_25–75%_ in never-smokers (*P* < 0.001 for quintile analysis, *P* = 0.001 for continuous variable analysis).

**Table 2:** Relationship between serum bilirubin and lung function according to smoking status

Lung function parameters	Bilirubin (Quintiles)						Bilirubin (Continuous)	
	Quintile I	Quintile 2	Quintile 3	Quintile 4	Quintile 5	*P* for Trend	ß ± SE	*P*-value
FEV_1_, L								
Continuous smoker	3.02 ± 0.62	3.17 ± 0.66	3.3 ± 0.62	3.44 ± 0.57	3.44 ± 0.6	0.048	0.09 ± 0.05	0.061
Former smoker	3.04 ± 0.69	3.21 ± 0.71	3.33 ± 0.64	3.43 ± 0.62	3.48 ± 0.61	0.193	0.02 ± 0.05	0.619
Never smoker	2.5 ± 0.51	2.57 ± 0.53	2.67 ± 0.55	2.77 ± 0.59	2.95 ± 0.64	< 0.001	0.10 ± 0.03	< 0.001
FVC, L								
Continuous smoker	3.95 ± 0.83	4.19 ± 0.79	4.3 ± 0.72	4.37 ± 0.68	4.38 ± 0.67	0.262	0.04 ± 0.06	0.441
Former smoker	3.94 ± 0.75	4.08 ± 0.79	4.21 ± 0.69	4.37 ± 0.69	4.32 ± 0.64	0.454	−0.02 ± 0.06	0.772
Never smoker	3.08 ± 0.6	3.16 ± 0.64	3.31 ± 0.98	3.39 ± 0.73	3.61 ± 0.79	0.018	0.10 ± 0.04	0.012
FEV_1_/FVC, %								
Continuous smoker	77.11 ± 7.71	76.42 ± 8.51	77.39 ± 7.63	78.54 ± 7.97	79.35 ± 7.48	0.205	1.02 ± 0.64	0.109
Former smoker	76.98 ± 9.09	77.85 ± 8.49	78.26 ± 8.01	78.88 ± 6.54	79.6 ± 7.27	0.516	0.52 ± 0.65	0.423
Never smoker	81.36 ± 6.68	81.62 ± 6.39	81.53 ± 6.37	81.99 ± 6.28	81.96 ± 6.16	0.027	0.76 ± 0.41	0.061
FEF_25–75%_, L/s								
Continuous smoker	2.88 ± 1.15	2.98 ± 1.23	3.16 ± 1.21	3.43 ± 1.34	3.97 ± 8.54	0.063	0.53 ± 0.39	0.176
Former smoker	2.83 ± 1.14	3.05 ± 1.18	3.26 ± 1.3	3.4 ± 1.19	3.53 ± 1.27	0.267	0.06 ± 0.10	0.534
Never smoker	2.77 ± 0.97	2.85 ± 0.91	2.97 ± 0.95	3.1 ± 1.02	3.28 ± 1.06	< 0.001	0.18 ± 0.06	0.001

Notes: Data are presented as the mean ± standard deviation. Bilirubin was analyzed across quintiles and as a continuous variable

P-value for linear trend analysis adjusted for age, sex, and body mass index

Abbreviations: *SE* standard error, *FEV*_*1*_ forced expiratory volume in 1 s, *FVC* forced vital capacity, *FEF*_*25–75%*_ forced expiratory flow between 25 and 75% of vital capacity

The results of multivariate regression analysis for the effect of serum bilirubin levels on repeated measures of lung function are described in Table 3. The FEV_1_ (estimated mean = 0.12, *P* < 0.001), FVC (estimated mean = 0.14, *P* < 0.001), and FEF_25–75%_ (estimated mean = 0.15, *P* < 0.001) were significantly associated with baseline bilirubin levels after adjustment for age, sex, BMI, and smoking status (Table 3). FEV_1_/FVC ratio tended to be related with baseline bilirubin level (estimated mean = 0.51, *P* = 0.0595).

**Table 3:** Multivariate regression analysis for the relationship between bilirubin and pulmonary lung function

Lung function parameters	Bilirubin	Bilirubin category				
		Quintile 1	Quintile 2	Quintile 3	Quintile 4	Quintile 5
FEV_1_, L						
Estimated mean (SE)	0.12(0.02)	ref	0.03(0.02)	0.06(0.02)	0.07(0.02)	0.11(0.02)
*P*-value	< 0.001		0.154	0.002	< 0.001	< 0.001
FVC, L						
Estimated mean (SE)	0.14(0.02)	ref	0.05(0.02)	0.10(0.02)	0.09(0.02)	0.13(0.02)
*P*-value	< 0.001		0.018	< 0.001	< 0.001	< 0.001
FEV_1_/FVC, %						
Estimated mean (SE)	0.51(0.27)	ref	−0.39(0.25)	−0.47(0.25)	0.22(0.25)	0.26(0.25)
*P*-value	0.059		0.113	0.055	0.371	0.307
FEF_25–75%_, L/s						
Estimated mean (SE)	0.15(0.04)	ref	0.01(0.05)	0.03(0.05)	0.11(0.05)	0.20(0.05)
*P*-value	< 0.001		0.857	0.575	0.022	< 0.001

Notes: Data were adjusted for age, sex, body mass index, and smoking status (current smoker, former smoker, and never-smoker)

Abbreviations: *SE* standard error, *FEV*_*1*_ forced expiratory volume in 1 s, *FVC* forced vital capacity, *FEF*_*25–75%*_ forced expiratory flow between 25 and 75% of vital capacity

### Bilirubin and lung function decline

**Fig. 2:**
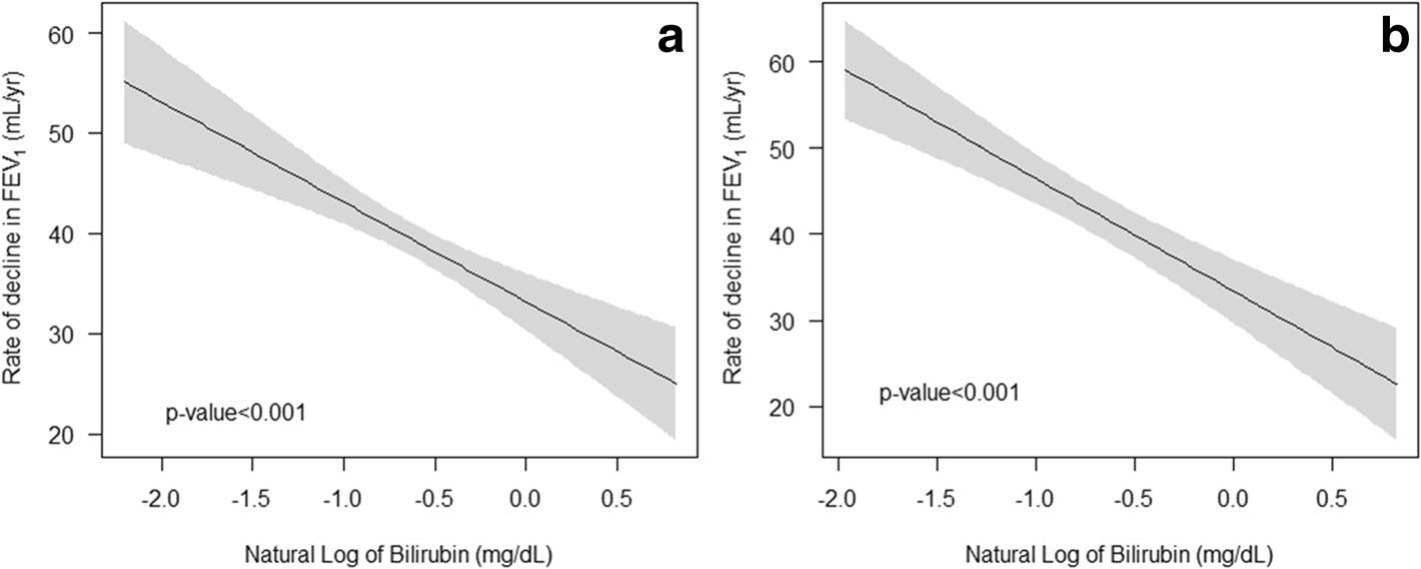
Average annual decline of FEV_1_ relative to the natural logarithm of serum bilirubin. Notes: Bilirubin is expressed in mg/dL in parentheses. **a** Univariate relationship (linear regression coefficienct = − 9.93) and **b** relationship adjusted for age, sex, BMI, FEV_1_ at baseline, and smoking status (current smoker, former smoker, and never-smoker)(linear regression coefficienct = − 13.09). Abbreviations: BMI, body mass index; FEV_1_, forced expiratory volume in 1 s

**Fig. 3:**
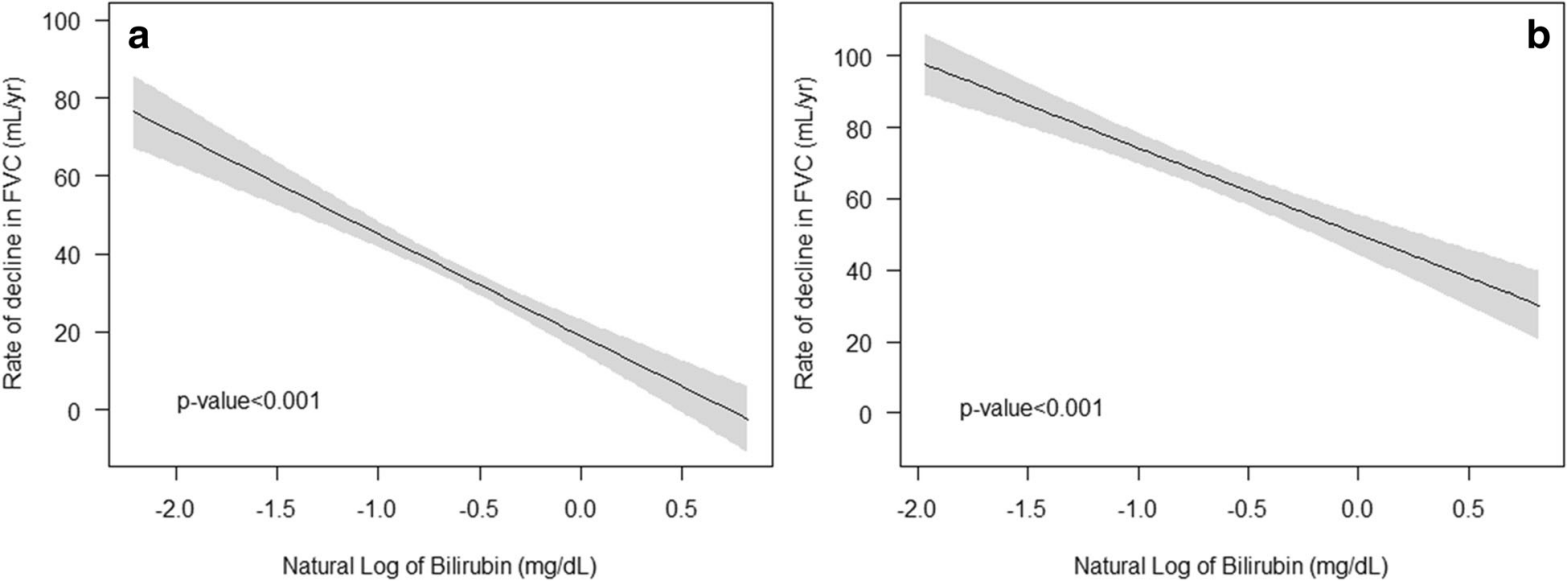
Average annual decline of FVC relative to the natural logarithm of serum bilirubin. Notes: Bilirubin is expressed in mg/dL in parentheses. **a** Univariate relationship (linear regression coefficienct = − 26.04) and **b** relationship adjusted for age, sex, BMI, FVC at baseline, and smoking status (current smoker, former smoker, and never smoker)(linear regression coefficienct = − 24.19). Abbreviations: BMI, body mass index; FVC, forced vital capacity

**Fig. 4:**
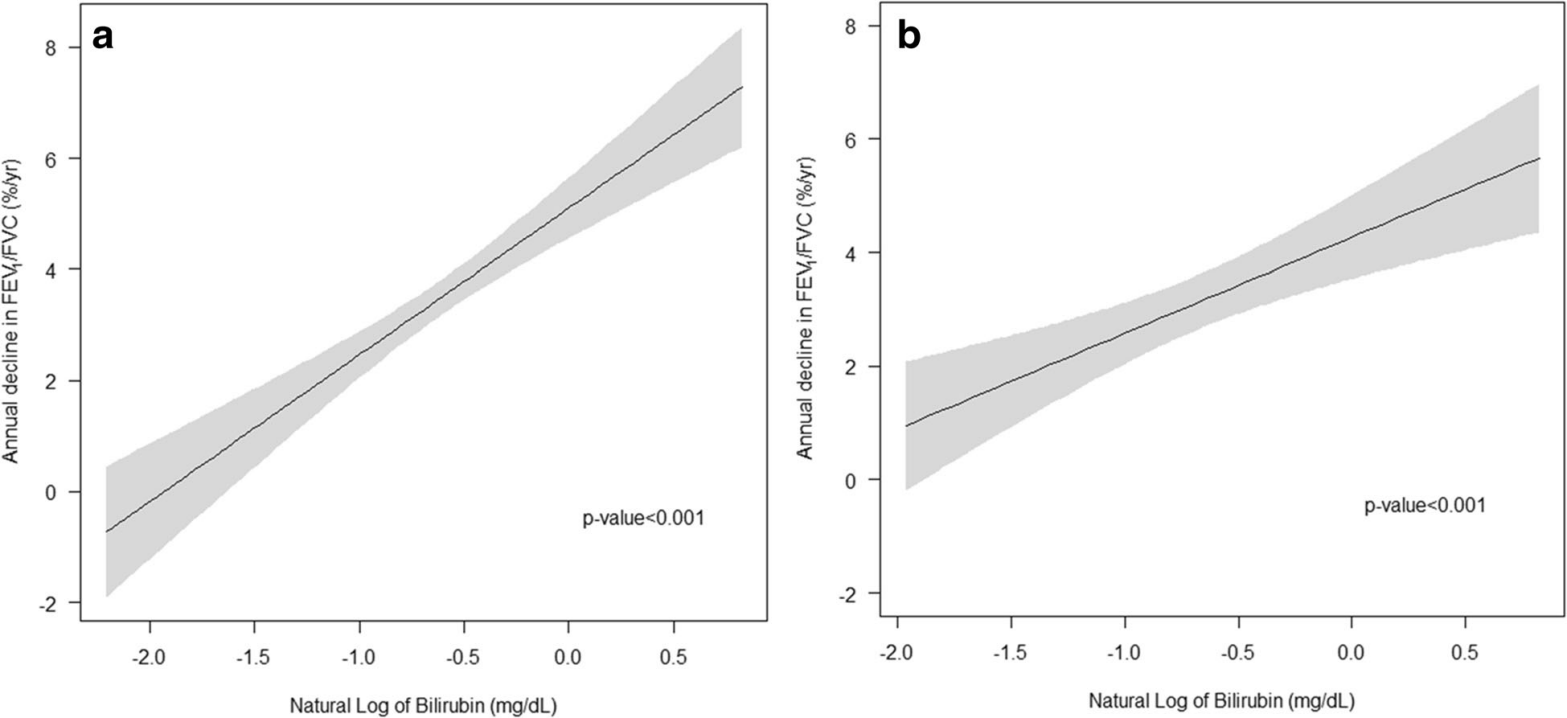
Average annual changes in FEV_1_/FVC ratio relative to the natural logarithm of serum bilirubin. Notes: Bilirubin is expressed in mg/dL in parentheses. **a** Univariate relationship (linear regression coefficienct = 2.64) and **b** relationship adjusted for age, sex, BMI, FEV_1_/FVC ratio at baseline, and smoking status (current smoker, former smoker, and never-smoker)(linear regression coefficienct = 1.69). Abbreviations: BMI, body mass index; FEV_1_, forced expiratory volume in 1 s; FVC, forced vital capacity

Figures 2, 3, and 4 show the relationship between bilirubin and the decline of lung function parameters. Higher bilirubin concentration was significantly related to reduced rate of annual decline in FEV_1_ (Fig. 2a) and FVC (Fig. 3a) (all *P* < 0.001). After adjustment for age, sex, BMI, baseline lung function, and smoking status, this inverse relationship persisted (all *P* < 0.001) (Figs. 2b and 3b). The annual change in FEV_1_/FVC ratio was positively associated with bilirubin concentration in univariate and multivariate adjusted analysis (all *P* < 0.001) (Fig. 4a, b).

## Discussion

In this population-based study, serum bilirubin was positively related with FEV_1_, FVC, and FEF_25–75%_. When stratified according to smoking status, these relationships were prominent in never-smokers. Furthermore, bilirubin concentration was inversely related to the rate of decline in FEV_1_ and FVC (mL/year). The annual decline in FEV_1_/FVC ratio was positively associated with bilirubin concentration. This is the first longitudinal study investigating the relationships between bilirubin levels and the changes of lung function parameters based on a general population-based cohort.

The beneficial effects of serum bilirubin on respiratory outcomes have been reported in several studies [5, 6, 12]. In a previous study by Temme et al., high serum bilirubin was associated with lower cancer mortality, including that of lung cancer [6]. In a study by Horsfall et al., relatively higher levels of bilirubin were associated with a lower risk of respiratory diseases such as lung cancer and COPD, and all-cause mortality among patients with normal-range bilirubin levels in primary care practices of United Kingdom [5]. It was the first large epidemiologic study to find that bilirubin concentration is negatively associated with COPD incidence. In a report by Brown et al., higher bilirubin was associated with a significantly lower hazard for acute exacerbation of COPD [12]. These results of previous studies suggest that bilirubin might have protective effects in lung tissues, possibly by counteracting subclinical inflammation. The results of our study are in line with previous results in that they showed the lung protective effect of bilirubin.

Only few studies about the relationship between bilirubin levels and lung function have been reported [7, 8]. Curjuric et al. showed that high bilirubin levels were significantly associated with higher FEV_1_/FVC and FEF_25–75%_ in the Swiss general population sample [7]. In that cross-sectional study, decreased serum bilirubin levels were associated with increased risk of COPD [7]. It was the only study that investigated the relationships between serum bilirubin and lung function in a healthy general population. For FEF_25–75%_, the results of that study were consistent with ours. The significant associations with FEF_25–75%_ might be related to the inverse relationship of bilirubin concentrations with small airway obstruction. However, both FEV_1_ and FVC were positively related to bilirubin concentration in our study, inconsistent with the results of Curjuric et al. in which bilirubin concentration was not significantly related to FEV_1_ or FVC. The differences in proportion of sex, distribution of smoking status, covariates adjusted may have resulted in different outcomes. In another study, Apperley et al. reported that serum bilirubin was positively related to FEV_1_ and negatively related to the annual decline in FEV_1_ in patients with mild to moderate COPD [8]. This was the only study that evaluated the relationships between serum bilirubin levels and the decline of FEV_1_ in patients with COPD [8]. We extended these results by demonstrating that serum bilirubin levels relate to FEV_1_ and FEV_1_ decline in a large community-based cohort of general population. Furthermore, we showed the relationships between serum bilirubin levels and other lung function parameters such as FVC, FEV_1_/FVC ratio, and FEF_25–75%_.

One study reported the relationships between serum inflammatory biomarkers, including total bilirubin, and COPD in never-smokers in the Korean population [13]. In that study, serum bilirubin concentration was not significantly associated with the presence of COPD in never-smokers [13]. However, the limitation of the study is that the sample size was small, and the relationship of bilirubin level with lung function was not assessed.

The possible related biological mechanisms of the role of bilirubin on lung function in COPD patients have been reported [14–17]. When heme is degraded to biliverdin by heme oxygenase, the production of bilirubin begins [14]. Biliverdin is subsequently reduced to bilirubin by biliverdin reductase [14]. Heme oxygenase-1 (HO-1), the inducible isoform of heme oxygenase expressed in type 2 pneumocytes and alveolar macrophages in the lung, has been reported to be up-regulated by oxidative stress and hypoxia [15, 16]. Genomic studies have found that the relative gene expression of heme oxygenase and biliverdin reductase is high in lung tissue [17]. The *HO-1* gene contains a variable number of GT nucleic acid repeats in its flanking region, and individuals with fewer GT repeats have higher serum bilirubin concentrations and a lower risk of COPD [18, 19].

In COPD, oxidative stress is affected by both endogenously produced oxidants and exogenous sources such as smoking. An animal study using animal model supported a protective effect of increased bilirubin against respiratory injury by environmental stressors [20]. In the study by Sedlak et al., bilirubin had a greater affinity in preventing oxidation of lipids, and inhibition of bilirubin synthesis resulted in significant increases in lipid peroxidation products [20].

In human lungs, it is reported that oxidant-antioxidant imbalance is associated with airways obstruction, and altered oxidant-antioxidant balance in patients with COPD increases in parallel with the severity of the disease [21]. Repine et al. reported that lipid peroxidation causes damage to multiple cell membrane components and impairs cell structure and permeability [22]. Patients with COPD have higher levels of lipid peroxidation products in sputum [23], and serum levels of these products are higher in patients with severe airflow limitation compared with those with moderate limitation [24]. Based on these results, we suggest that bilirubin might protect the lungs by inhibiting lipid peroxidation [21–24].

To our knowledge, this is the first longitudinal study that analyzed the relationships between bilirubin levels and changes in lung function parameters in the healthy general population. The study was conducted in a prospective, large cohort using various spirometric measures. However, the study also has several limitations. First, the follow-up period was relatively short, yet the Ansung-Ansan cohort study is still ongoing. We expect to be able to analyze the data further with longer follow-up. Second, we used pre-bronchodilator spirometric values instead of post-bronchodilator values. Third, serum bilirubin concentration was only measured at baseline and it is unsure if bilirubin level was stable during the follow-up period. Therefore, the relationships between the changes of bilirubin concentration and lung function decline could not be analyzed. Lastly, the information on alcohol consumption, previous drug intake, or diet which can affect bilirubin level was not fully investigated in this cohort study.

## Conclusions

We found significant positive associations of serum bilirubin levels with FEV_1_, FVC, and FEF_25–75%_ in the general population, especially in never-smokers after adjustment of age, sex, BMI, and smoking status. Furthermore, serum bilirubin concentration was also significantly related to the annual changes in FEV_1_, FVC, and FEV_1_/FVC ratio. Further research is needed to investigate causal associations between bilirubin levels and lung function.

## Abbreviations

BMI: Body mass index
COPD: Chronic obstructive pulmonary disease
FEF_25–75%_: Mean forced expiratory flow between 25 and 75% of FVC
FEV_1_: Forced expiratory volume in 1 s
FVC: Forced vital capacity
SD: Standard deviation

## References

[1] Kronenberg F (2010). Association of bilirubin with cardiovascular outcomes: more hype than substance?. Circ Cardiovasc Genet.

[2] Stocker R, Yamamoto Y, McDonagh AF, Glazer AN, Ames BN (1987). Bilirubin is an antioxidant of possible physiological importance. Science.

[3] Schwertner HA, Vitek L (2008). Gilbert syndrome, UGT1A1*28 allele, and cardiovascular disease risk: possible protective effects and therapeutic applications of bilirubin. Atherosclerosis.

[4] Lin JP, Vitek L, Schwertner HA (2010). Serum bilirubin and genes controlling bilirubin concentrations as biomarkers for cardiovascular disease. Clin Chem.

[5] Horsfall LJ, Rait G, Walters K, Swallow DM, Pereira SP, Nazareth I (2011). Serum bilirubin and risk of respiratory disease and death. JAMA.

[6] Temme EH, Zhang J, Schouten EG, Kesteloot H (2001). Serum bilirubin and 10-year mortality risk in a Belgian population. Cancer Causes Control.

[7] Curjuric I, Imboden M, Adam M, Bettschart RW, Gerbase MW, Kunzli N (2014). Serum bilirubin is associated with lung function in a Swiss general population sample. Eur Respir J.

[8] Apperley S, Park HY, Holmes DT, Man SF, Tashkin D, Wise RA (2015). Serum bilirubin and disease progression in mild COPD. Chest.

[9] Baik I, Cho NH, Kim SH, Han BG, Shin C (2011). Genome-wide association studies identify genetic loci related to alcohol consumption in Korean men. Am J Clin Nutr.

[10] Leem AY, Park B, Kim YS, Jung JY, Won S (2018). Incidence and risk of chronic obstructive pulmonary disease in a Korean community-based cohort. Int J Chron Obstruct Pulmon Dis.

[11] Ha KH, Kim HC, Park S, Ihm SH, Lee HY (2014). Gender differences in the association between serum gamma-glutamyltransferase and blood pressure change: a prospective community-based cohort study. J Korean Med Sci.

[12] Brown KE, Sin DD, Voelker H, Connett JE, Niewoehner DE, Kunisaki KM (2017). Serum bilirubin and the risk of chronic obstructive pulmonary disease exacerbations. Respir Res.

[13] Lee H, Hong Y, Lim MN, Bak SH, Kim MJ, Kim K, et al. Inflammatory biomarkers and radiologic measurements in never-smokers with COPD: a cross-sectional study from the CODA cohort. Chron Respir Dis. 2017; 10.1177/1479972317736293.1479972317736293. 10.1177/1479972317736293 PMC5958470 29117798

[14] Chen W, Maghzal GJ, Ayer A, Suarna C, Dunn LL, Stocker R (2018). Absence of the biliverdin reductase-a gene is associated with increased endogenous oxidative stress. Free Radic Biol Med.

[15] Carter EP, Garat C, Imamura M (2004). Continual emerging roles of HO-1: protection against airway inflammation. Am J Physiol Lung Cell Mol Physiol.

[16] Fredenburgh LE, Perrella MA, Mitsialis SA (2007). The role of heme oxygenase-1 in pulmonary disease. Am J Respir Cell Mol Biol.

[17] Burren OS, Adlem EC, Achu_than P, Christensen M, Coulson RM, Todd JA (2011). T1DBase: update 2011, organization and presentation of large-scale data sets for type 1 diabetes research. Nucleic Acids Res.

[18] D'Silva S, Borse V, Colah RB, Ghosh K, Mukherjee MB (2011). Association of (GT)n repeats promoter polymorphism of heme oxygenase-1 gene with serum bilirubin levels in healthy Indian adults. Genet Test Mol Biomarkers.

[19] Yamada N, Yamaya M, Okinaga S, Nakayama K, Sekizawa K, Shibahara S (2000). Microsatellite polymorphism in the heme oxygenase-1 gene promoter is associated with susceptibility to emphysema. Am J Hum Genet.

[20] Sedlak TW, Saleh M, Higginson DS, Paul BD, Juluri KR, Snyder SH (2009). Bilirubin and glutathione have complementary antioxidant and cytoprotective roles. Proc Natl Acad Sci U S A.

[21] Singh S, Verma SK, Kumar S, Ahmad MK, Nischal A, Singh SK (2017). Evaluation of oxidative stress and antioxidant status in chronic obstructive pulmonary disease. Scand J Immunol.

[22] Repine JE, Bast A, Lankhorst I (1997). Oxidative stress in chronic obstructive pulmonary disease. Oxidative Stress Study Group Am J Respir Crit Care Med.

[23] Nowak D, Kasielski M, Antczak A, Pietras T, Bialasiewicz P (1999). Increased content of thiobarbituric acid-reactive substances and hydrogen peroxide in the expired breath condensate of patients with stable chronic obstructive pulmonary disease: no significant effect of cigarette smoking. Respir Med.

[24] Kluchova Z, Petrasova D, Joppa P, Dorkova Z, Tkacova R (2007). The association between oxidative stress and obstructive lung impairment in patients with COPD. Physiol Res.

